# Whole rat stomach decellularisation using a detergent-enzymatic protocol

**DOI:** 10.1007/s00383-018-4372-8

**Published:** 2018-11-16

**Authors:** Elisa Zambaiti, Federico Scottoni, Eleonora Rizzi, Simone Russo, Koichi Deguchi, Simon Eaton, Alessandro F. Pellegata, Paolo De Coppi

**Affiliations:** 1 Stem Cell and Regenerative Medicine Section, DBC, UCL, Great Ormond Street Institute of Child Health, University College of London, Surgery Offices, 30 Guilford Street, London, WC1N 1EH UK; 2 Specialist Neonatal and Paediatric Surgery, Great Ormond Street Hospital for Children NHS Foundation Trust, London, UK

**Keywords:** Stomach, Decellularisation, Tissue engineering, Extracellular matrix, Scaffold, Microgastria, Gastrectomy

## Abstract

**Background:**

Conditions leading to reduced gastric volume are difficult to manage and are associated to poor quality-of-life. Stomach augmentation using a tissue-engineered stomach is a potential solution to restore adequate physiology and food reservoir. Aim of this study was to evaluate the decellularisation of whole rat stomach using a detergent-enzymatic protocol.

**Methods:**

Stomachs harvested from rats were decellularised through luminal and vascular cannulation using 24-h detergent-enzymatic treatment and completely characterized by appropriate staining, DNA and Extracellular matrix -component quantifications.

**Results:**

The detergent-enzymatic protocol allows a complete decellularisation of the gastric tissue, with a complete removal of the DNA with two cycles as confirmed by both quantifications and histological analysis. Extracellular matrix components, collagen, fibronectin, laminin and elastin, were optimally preserved by the treatment, while glycosaminoglycans were reduced.

**Conclusion:**

Gastric tissue can be efficiently decellularised. Scaffolds retained original structure and important components that could enhance integration with other tissues for in vivo transplant. The use of naturally derived material could be potentially considered for the treatment of both congenital and acquired conditions.

## Introduction

Congenital and acquired conditions can lead to a reduced volume of the stomach. Congenital microgastria is a rare condition first reported in the 1800s. Less than 50 cases have been reported in the literature so far. Gastric tissue is widely used in the surgical treatment of congenital and acquired childhood diseases involving the oesophagus. Whether to achieve correction of long gap oesophageal atresia or to treat oesophageal stenosis following caustic ingestion, part of the stomach can be tubulised and used as substitute for the unavailable or unusable oesophagus [1]. In adults, gastric tissue can also be lost following bariatric surgery such as gastric sleeve surgery or after partial gastric resection due to cancer. These conditions, together with the rare congenital pathology known as microgastria [2], could cause a high morbidity due to reduced gastric volume and related loss of digestive and reservoir function. Following total or partial gastrectomy, dumping syndrome and reduced intestinal adsorption of vitamins are indeed to be kept in mind dealing with children since they can influence life-long quality-of-life [3].

Gastric augmentation could potentially reduce the symptoms described above; however, current strategies involve complex surgical reconstruction using the intestine and achieve sub-optimal results. Roux–en–Y (Hunt-Lawrence pouch) jejunal loops have been recommended to increase gastric volume in microgastria, but leads to patients showing highly abnormal motility with hypomotile or obstructive patterns.

It would be ideal if gastric tissue could be engineered in an autologous setting. Regenerative medicine is an emerging field in science showing the possibility of healing and regenerate damaged or missing tissue. Decellularised tissue in particular seems to have the potential to be used for the replacement of damaged tissue or organs, and we have successfully transplanted engineered cadaveric trachea to substitute children airway. There are several advantages on using decellularised scaffolds which preserve the native extracellular-matrix (ECM) architecture, its composition and all the biological cues which are necessary for regeneration.

To date, there have been no study published using decellularised gastric tissue as xenogenic material for stomach augmentation. Aim of this paper is to develop a decellularisation protocol to efficiently create a scaffold suitable for stomach augmentation.

## Materials and methods

### Tissue isolation

Whole stomachs were harvested from female and male Sprague Dawley rats, weighing approximately 200–250 g. After sacrifice, the abdominal wall was sterilized with 70% ethanol and a midline incision was performed to expose the abdominal cavity. Celiac artery was cannulated with a 27G cannula (Introcan Certo, B. Braun Medical AG, Germany) secured in place with a 3–0 silk suture (Ethicon, UK). Hepatic artery was ligated and portal vein sectioned. The stomach was harvested and barbed to luer-lock connectors (Cole Parmer, US) were used to cannulate cardias and pylorus.

Stomachs were decellularised using a detergent-enzymatic treatment (DET) as previously described. Both the vasculature and the organ lumen were perfused with a 1 ml/min flow rate using a Masterflex L/S variable speed roller pump (Masterflex, US). Each DET cycle was consisting of: 24 h perfusion with deionized water (18.2 MU/cm), 4 h with 4% sodium deoxycholate (Sigma, UK), 1 h with deionized water, 3 h 22,5 mg/l DNase-I in 0,9% sodium chloride (NaCl, Sigma, UK) and 1,11 g/l calcium chloride (CaCl_2_, Sigma, UK). All organs after DET were preserved at 4 °C, in PBS (Gibco, UK) and antibiotics (Penicillin/streptomycin 1%) and irradiated to eliminate all contaminants.

### Histological evaluation

To allow histologic evaluation, samples were fixed in 4% paraformaldehyde (PFA; Sigma, UK). Afterward they have been dehydrated in ethanol scale, paraffin-embedded and sectioned with a 7 µm thickness.

Tissue slides were stained with Haematoxylin and Eosin, Masson’s Trichrome (RAL Diagnostic) Picro-sirius Red (Abcam), Elastica Van Gieson (EMD Millipore corporation) and Alcian Blue (Sigma–Aldrich) stains. Appropriate positive controls have been used to ensure that histological stains were correctly performed. For all histological samples three biological and technical replicates have been assessed using standard Haematoxylin and Eosin prior to any special stains to ensure homogeneity.

### Immunohistochemistry

Immunohistochemistry (IHC) was performed with DAKO Dual Link System-HRP DAB+ (Dako North America). Staining was performed on paraffin-embedded slides, after antigen retrieval. Permeabilization was performed with 25 min incubation in 0.5% PBS Triton X at room temperature and quenching of endogenous peroxidase activity using 3% H_2_O_2_ in PBS for 10 min. Later, the slides were incubated for 20 min with one drop of the blocking agent Dual Endogenous Enzyme Block and then incubated overnight with primary antibodies diluted in Antibody Diluent provided as listed. Primary antibodies against fibronectin (Santa Cruz, SC59826), laminin (Abcam, AB11575) and collagen IV (Abcam, AB6586) were used at dilutions of 1:100 in BSA 1%. The following day, slides were washed rapidly with 0.05% tween in PBS and out 30 min to incubate with Labelled Polymer-HRP (Dako Kit). Finally, slides were developed with DAB Permanent Stain Solution, counterstained with Hematoxylin and mounted. Appropriate fresh tissue and no primary antibody controls were used to ensure appropriate tissue positivity.

Paraffin-embedded slides were rehydrated and underwent quenching of endogenous peroxidase activity using 1% H2O2 (Sigma, UK). Following quenching, antigen retrieval was performed with pepsin (Sigma, UK) at a concentration of 1 mg/ml in 1N HCl (Sigma, UK), at 37 °C for 60 min. Appropriate fresh tissue and no primary antibody controls were used to ensure appropriate tissue positivity.

### Characterisation of decellularised tissue

The DNA content of decellularised tissues was assessed using the Qiagen DNeasy Blood and Tissue kit (Qiagen, Valencia, CA) following manufacturer’s protocol. Briefly, normal and decellularised tissues were enzymatically lysed using proteinase K and then passed through a selective DNA binding membrane. Purified DNA elute was analysed spectrophotometrically (Nanodrop, Thermo Scientific, US) and normalised by weight. Optical densities at 260 nm and 280 nm were used to estimate the purity and yield of nucleic acids.

To further confirm staining results, we also performed ECM components quantifications.

The collagen content was quantified using the Total collagen assay (QuickZyme, UK). Samples were homogenized and collagen was solubilized in 0.5 M acetic acid. Extracts were incubated with Sirius red dye, and absorbance was determined at 555 nm with a microplate reader (Tecan Infinity). The elastin content was quantified using the FASTIN elastin assay (Biocolor, UK). After homogenization, elastin was solubilized in 0.25 M oxalic acid to ensure complete extraction of elastin. Extracts were incubated with 5,10,15,20-tetraphenyl-21H,23H-porphine tetrasulfonate (TPPS) dye, and absorbance was determined at 555 nm spectrophotometrically (Tecan Infinity, US). The sulfated glycosaminoglycan (GAG) content was quantified using the Blyscan GAG Assay Kit (Biocolor, UK). In brief, 50 mg of minced wet tissue was weighed and placed in a micro-centrifuge tube containing 1 ml of Papain digestion buffer and incubated in a water bath at 65 °C for 18 h, with occasional tube removal and vortexing. Aliquots of each sample were mixed with 1,9-dimethyl-methylene blue dye and reagents from the kit. The absorbance at 636 nm was measured using a microplate reader (Tecan Infinity). Collagen, elastin and GAGs concentrations from a standard curve were used to calculate the content of each ECM component in the tissue. For all quantifications at least three biological and technical replicates were obtained.

### Statistic

*P* values are described in the figure legends. Mann–Whitney and Kruskal–Wallis tests were used. The investigators were blinded during experiments.

## Results

**Fig. 1:**
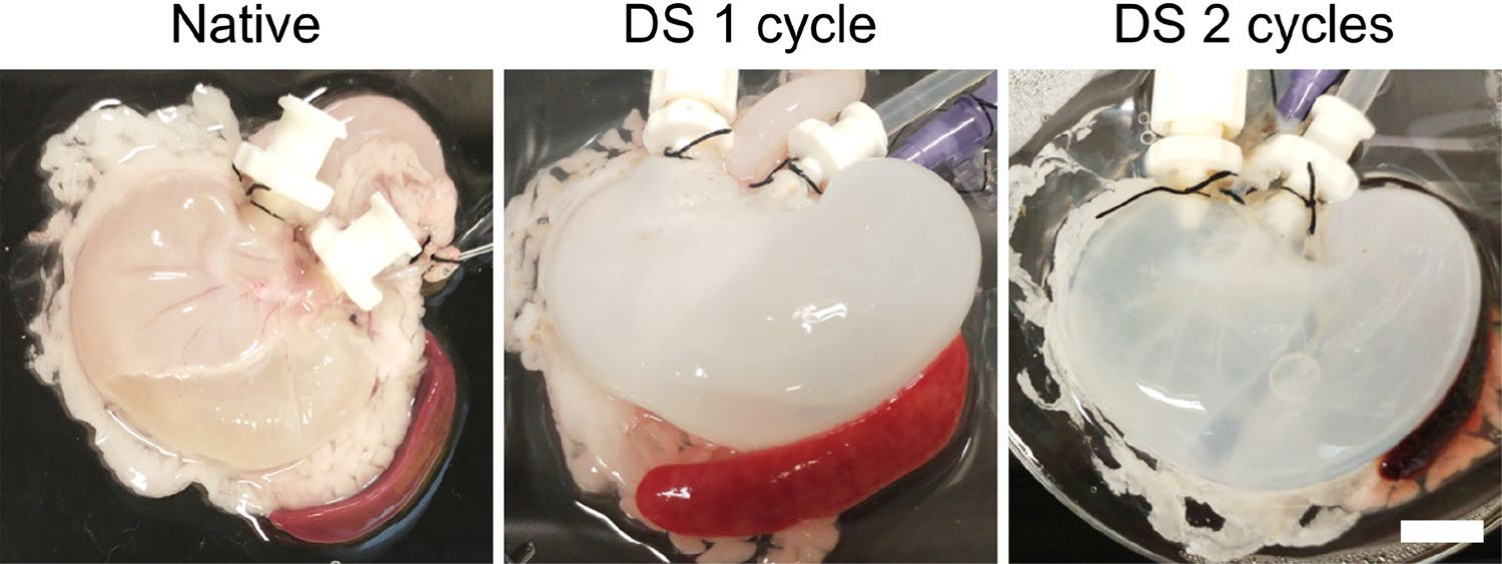
Macroscopic appearance of decellularised rat stomachs after 1 and 2 decellularisation cycles showing preservation of the macroscopic structure compared to the native organ (scale bar 1 cm)

**Fig. 2:**
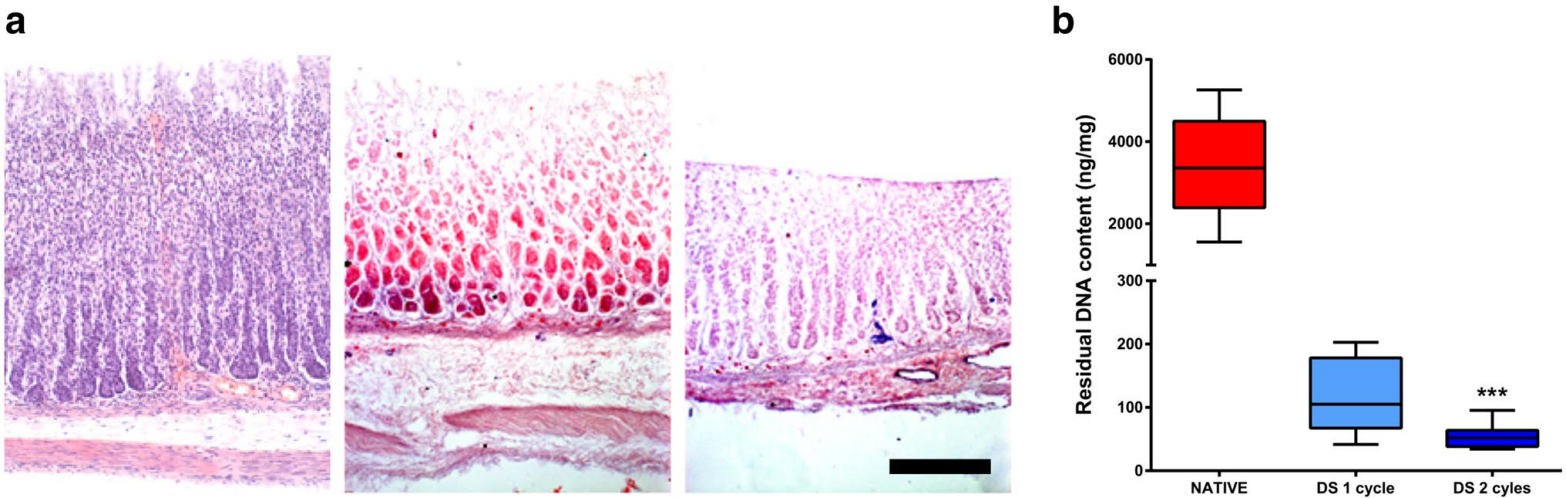
**a** Haematoxylin and eosin staining showing preservation of the micro structure throughout the whole tissue thickness in decellularised rat stomachs after 1 and 2 decellularisation cycles compared to the native organ (scale bar 100 µm). **b** Residual DNA content showing that two decellularisation cycles are required to reach an efficient decellularisation

Stomachs were decellularised using either 1 or 2 detergent-enzymatic decellularisation cycles. Macroscopic appearance of the whole organ shows preservation of the structure without any evident failure even after two decellularisation cycles (Fig. 1). Histologic appearance showed removal of cells and preservation of the microstructure. The mucosal glandular pattern is preserved and the muscle layer retained ECM fibres orientation and vessels (Fig. 2a). DNA quantification was performed to validate cell removal, and it showed that two decellularisation cycles were required to achieve a significant reduction in the DNA content (Fig. 2b).

**Fig. 3:**
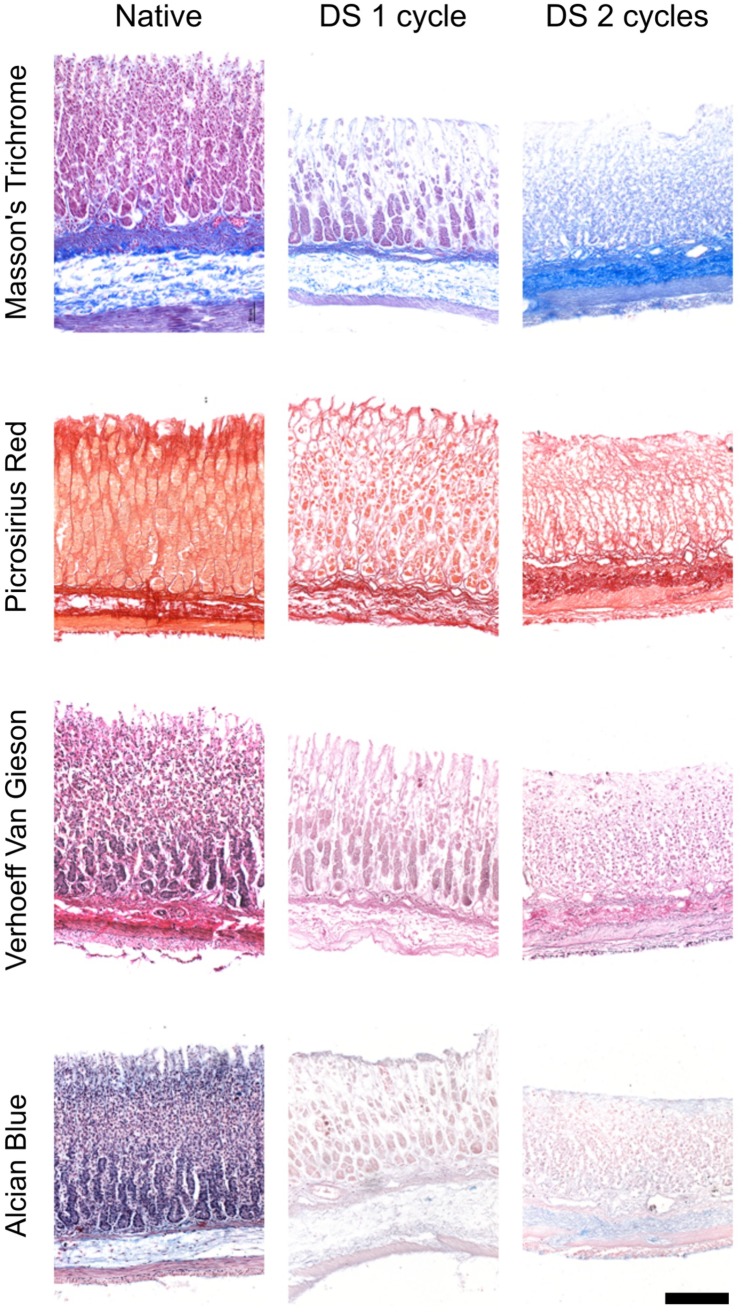
Principal extracellular matrix components distribution is maintained as shown by respective histological markers (scale bar 100 µm)

**Fig. 4:**
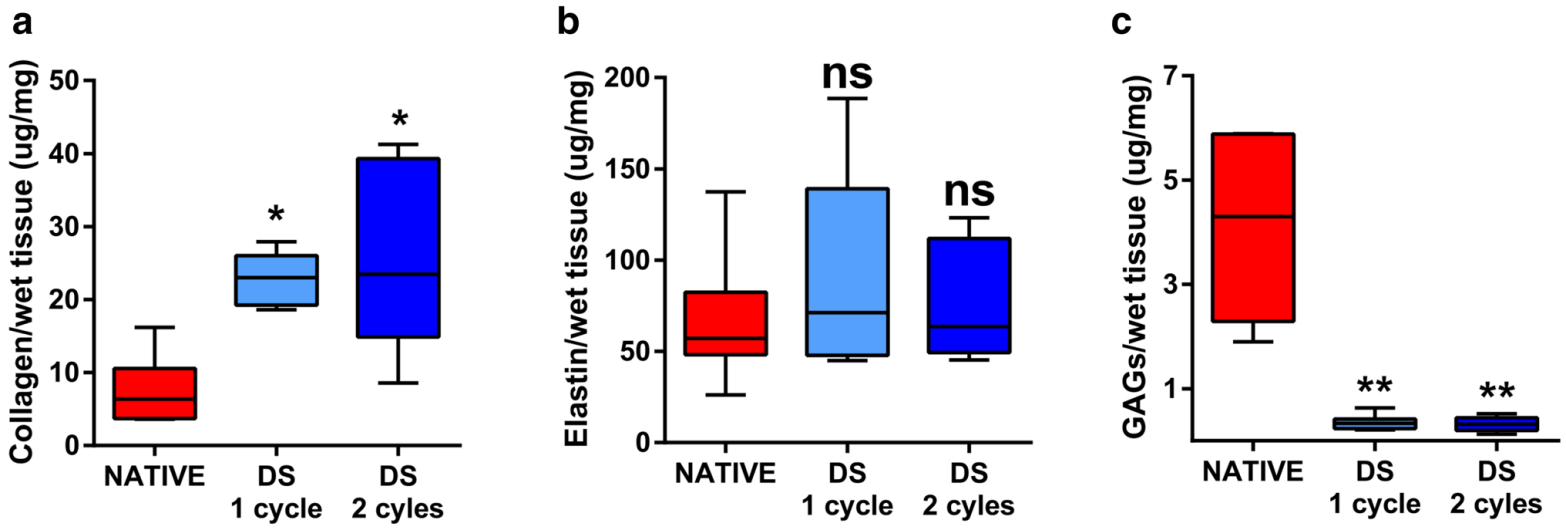
**a** Collagen quantification shows an increase in the relative collagen content. **b** Elastin quantification shows preservation of the relative elastin content. **c** Glycosaminoglycans (GAG) quantification shows a reduction in the relative GAGs content

**Fig. 5:**
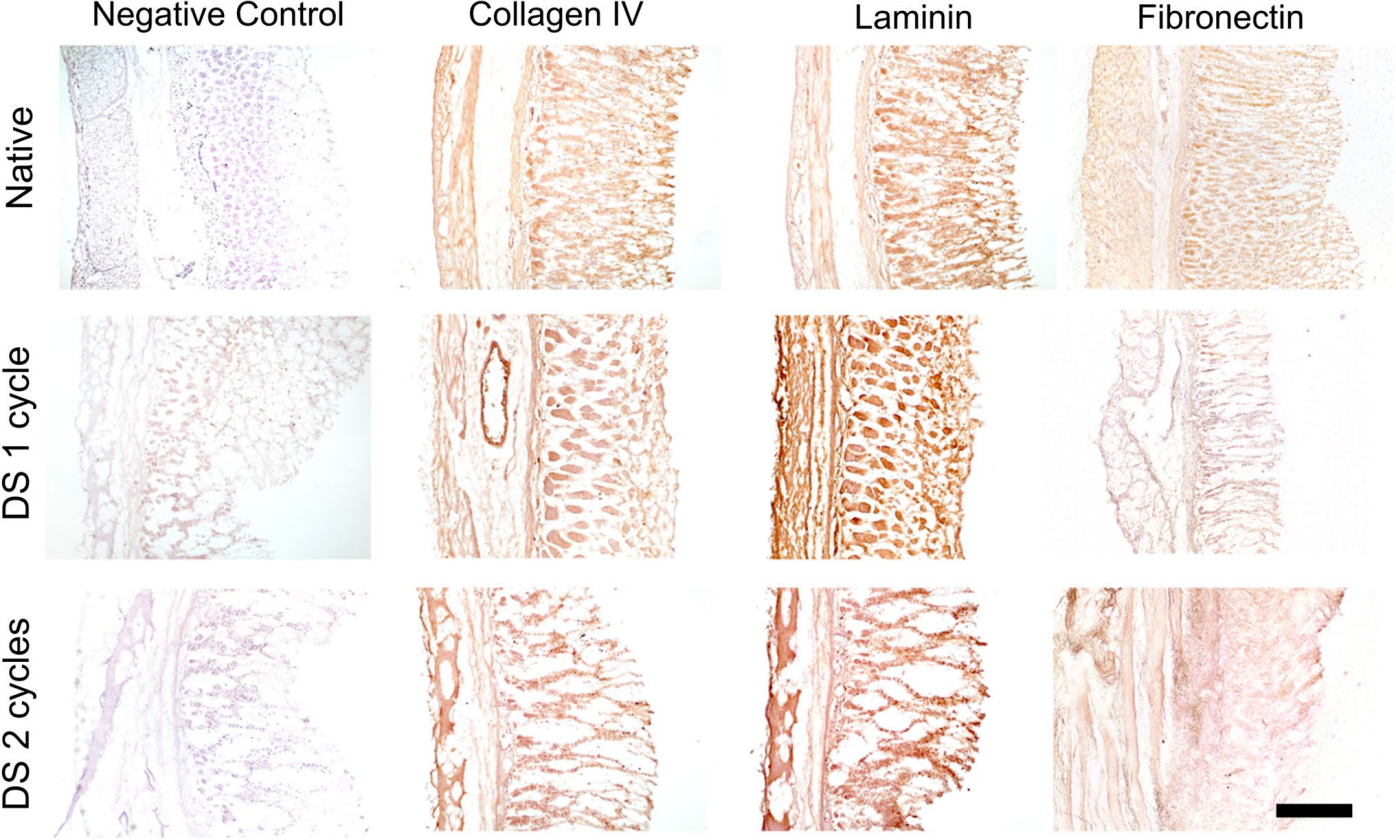
Immunohistochemistry staining of stomachs which were decellularised using 1 and 2 cycles compared to native tissue showing that collagen IV, laminin and fibronectin are maintained after the decellularisation process and their distribution in the tissue is preserved (scale bar 100 µm)

ECM components were assessed using staining and quantifications. Overall the different ECM components retained their distribution as confirmed by the specific staining, in particular Masson Trichrome confirmed cell removal (Fig. 3). Quantification of collagen revealed an increase in its relative content (Fig. 4a) while elastin was preserved (Fig. 4b) and a reduction was observed in glycosaminoglycans (Fig. 4c). Immunostaining demonstrated that collagen type IV structure as fine strands in the muscular layer, as well as in the mucosa was preserved even after two decellularisation cycles as well as laminin and fibronectin which retained their pattern throughout the whole stomach thickness (Fig. 5).

## Discussion

In this study we demonstrate that whole rat stomachs can be decellularised using a detergent-enzymatic protocol which preserves the micro- and macro-structure of the extracellular matrix. Decellularisation has been investigated over the last few years on almost every tissue with the aim of producing suitable scaffold for tissue engineering and regenerative medicine purposes [4]. While removing cells from tissues has been explored since the seventies [5], it is only in the last decade that ECM derived from tissues and organs has regained major attention for the implication in clinical translation [6]. Acellular matrices obtained by a detergent-enzymatic method [5] can be used to regenerate various both relatively simple tissues such as oesophagus [7] trachea [8, 9], skeletal muscle [10, 11], and small bowel [12], but they can also be used to prepare matrices from complex organs such as liver [13] and lungs [14]. Decellularised tissues have also reached clinical application for some specific tissues, as for the trachea [15]. Interestingly, decellularised tissue may also be used in the context of xenogenic transplantation where they have proved to maintain their pro-regenerative potential [16].

However, few attempts have been made so far to deliver a full decellularised stomach to be used for tissue engineering purposes [17–20]. Besides being used to repair oesophagus or intestine, decellularised stomach could potentially provide an easy-to-use and safe scaffold for stomach augmentation to treat microgastria and post-gastrectomy syndromes. Definitive surgery in the form of a Hunt-Lawrence (HL) jejunal pouch for gastric augmentation is a complex surgery associated to complications such as dumping syndrome, leakage and obstruction [21, 22]. Despite pouch augmentation, children with microgastria have generally feeding problems, resulting in failure to thrive and growth retardation [23]. Similarly, adults receiving partial gastrectomy following cancer resection or undergoing bariatric surgery needs frequent small feeding and may also require jejunal feeding which is associated to a lower quality-of-life [24].

The results that we have obtained in this study pose strong basis for a future use of decellularised scaffold in the context of stomach augmentation. Indeed, we applied a gentle detergent-enzymatic protocol which we have used before in the context of other tissues [12, 14, 25, 26]. This gentle protocol is able to properly remove the cellular component, as demonstrated by the absence of nuclei in the hematoxylin–eosin staining and by the DNA quantification. Alongside cell removal, this protocol is able to maintain tissue structure, which is of crucial importance for tissue re-cellularisation, since the ECM structure and composition drive the regeneration process in decellularised tissues [27]. In particular, we have demonstrated that the main components of the extracellular matrix are overall conserved both structurally and on their composition with some variations. Specifically, collagen quantification showed an increase in the relative content, which is due to the fact that, by removing cells, the ratio between the different ECM components change in respect to the overall tissue weight, in particular for collagen which is the most abundant one as previously described for other tissues [28]. Beside collagen, elastin ratio was maintained, of note, preservation of collagen and elastin is an important feature to maintain the biomechanical response of the tissue once transplanted. Glycosaminoglycans (GAG) quantity dropped instead. This is related to the fact that GAGs are highly present not only on the ECM, but also in cells which have been removed by the decellularisation process.

In conclusion, this work highlights the possibility of decellularising the whole stomach in rat using a simple enzymatic treatment. Applying decellularisation to gastric tissue may have clinical implication for the treatment of complex diseases such as microgastria. Future work will be dedicated to the translation of this protocol to large animal models for the delivering a gastric scaffold without cellular remnants that maintains tissue structure and ECM component.
